# Evaluation of tumor recurrences after radical prostatectomy using 18F-Choline PET/CT and 3T multiparametric MRI without endorectal coil: a single center experience

**DOI:** 10.1186/s40644-016-0099-8

**Published:** 2016-12-07

**Authors:** Felipe Couñago, Manuel Recio, Antonio Maldonado, Elia del Cerro, Ana Aurora Díaz-Gavela, Israel J. Thuissard, David Sanz-Rosa, Francisco José Marcos, Karmele Olaciregui, María Mateo, Laura Cerezo

**Affiliations:** 1 Department of Radiation Oncology, Hospital Universitario Quiron Madrid, Calle Diego de Velazquez, 1, 28223 Pozuelo de Alarcón Madrid, Spain; 2 Department of Radiology, Hospital Universitario Quiron, Madrid, Spain; 3 Department of Nuclear Medicine, Hospital Universitario Quiron, Madrid, Spain; 4 School of Doctoral Studies and Research, Universidad Europea de Madrid, Madrid, Spain; 5 Clinical Department, School of Biomedical Sciences, Universidad Europea de Madrid, Madrid, Spain; 6 Hospital Universitario Quiron, Madrid, Spain; 7 Department of Radiation Oncology, Hospital Universitario La Princesa, Madrid, Spain

**Keywords:** Prostate cancer, Radical prostatectomy, Biochemical failure, Multiparametric MRI, 18F-Choline PET/CT

## Abstract

**Background:**

To evaluate and compare the utility of 18F-fluorocholine (18F-CH) PET/CT versus 3-Tesla multiparametric MRI (mpMRI) without endorectal coil to detect tumor recurrences in patients with biochemical relapse following radical prostatectomy (RP). Secondarily, to identify possible prognostic variables associated with mpMRI and 18F-CH PET/CT findings.

**Methods:**

Retrospective study of 38 patients who developed biochemical recurrence after RP between the years 2011 and 2015 at our institution. PET/CT and mpMRI were both performed within 30 days of each other in all patients. The PET/CT was reviewed by a nuclear medicine specialist while the mpMRI was assessed by a radiologist, both of whom were blinded to outcomes.

**Results:**

The median prostate-specific antigen (PSA) value pre-MRI/PET-CT was 0.9 ng/mL (interquartile range 0.4–2.2 ng/mL). There were no differences in the detection rate between 18F-CH PET/CT and mpMRI for local recurrence (LR), lymph node recurrence (LNR) and bone metastases (BM). Separately, mpMRI and 18F-CH PET/CT were positive for recurrence in 55.2% and 52.6% of cases, respectively, and in 65.7% of cases when findings from both modalities were considered together. The detection of LR was better with combined mpMRI and choline PET/CT versus choline PET/CT alone (34.2% vs 18.4%, *p* = 0.04). Salvage treatment was modified in 22 patients (57.8%) based on the imaging findings. PSA values on the day of biochemical failure were significantly associated with mpMRI positivity (adjusted odds ratio (OR): 30.9; 95% confidence interval (CI): 1.5–635.8). Gleason score > 7 was significantly associated with PET/CT positivity (OR: 13.9; 95% CI: 1.5–125.6). A significant association was found between PSA doubling time (PSADT) (OR: 1.3; 95% CI: 1.0–1.7), T stage (OR: 21.1; 95% CI: 1.6–272.1), and LR.

**Conclusions:**

Multiparametric MRI and 18F-CH PET/CT yield similar detection rates for LR, LNR and pelvic BM. The combination of both imaging techniques provides a better LR detection versus choline PET/CT alone. The initially planned salvage treatment was modified in 57.8% of patients due to imaging findings. In addition to PSA values, Gleason score, T stage, and PSADT may provide valuable data to identify those patients that are most likely to benefit from undergoing both imaging procedures.

**Electronic supplementary material:**

The online version of this article (doi:10.1186/s40644-016-0099-8) contains supplementary material, which is available to authorized users.

## Background

After radical prostatectomy (RP), 20–50% of patients with prostate cancer (PCa) will develop a tumor recurrence within ten years [1]. In patients with recurrent disease, accurate identification of the site of recurrence is crucial because the type of salvage treatment administered depends on whether the patient presents pelvic recurrence: local recurrence (LR) with or without pelvic lymph node recurrence (LNR) and/or bone metastases (BM), or extrapelvic distant metastases [2]. Currently, multiparametric magnetic resonance imaging (mpMRI) and 11C or 18F-choline (18F-CH) PET/CT are considered the diagnostic imaging tests of choice in this patient population [2]. However, in recent years prostate-specific membrane antigen (PSMA)-ligand imaging has shown promising results in detecting recurrences after RP, and for this reason use of this approach has become increasingly common [for a Review, see 3].

Multiparametric MRI has proven useful in the detection of LR in the prostate gland, even in patients with low prostate-specific antigen (PSA) levels (<0.5 ng/mL) [4], although few studies have assessed the value of mpMRI to LNR or pelvic bone BM [2, 5]. By contrast, 18F-CH PET/CT has proven effective in detecting LNR and BM after RP, primarily in patients with PSA >1 ng/mL or with PSA doubling time (PSADT) < 6 months [6]. The utility of 18F-CH PET/CT to detect LR has received only scant attention [1, 7].

Panebianco et al. compared mpMRI with endorectal coil, dynamic contrast enhanced (DCE), and spectroscopy to 18F-CH PET/CT [7], finding that mpMRI was more reliable at detecting LR. Another study recently compared mpMRI with endorectal coil to 11C-choline PET/CT to detect pelvic recurrences [8], finding that mpMRI was more accurate in detecting LR whereas 11C-PET/CT yielded better results in detecting LNR. Both methods presented similar detection rates for bone metastases. Other studies evaluating the role of PET/CT and mpMRI in PCa staging have found that the sensitivity of mpMRI to detect nodal metastases is similar to, or slightly lower than, that of choline PET/CT [9–11]. A recent meta-analysis found that mpMRI had a higher sensitivity and a lower specificity than choline PET/CT in the diagnosis of BM [12]. To our knowledge, no studies have been conducted to date to directly compare 3T-mpMRI without endorectal coil with diffusion weighted imaging (DWI) and DCE to 18F-CH PET/CT in the detection of pelvic recurrences after RP.

Given this context, the aim of the present study was to compare 18F-CH PET/CT to 3T mpMRI with a phased-array torso coil to determine their relative capabilities to detect pelvic LR, LNR, and BM after RP and to assess their impact on salvage therapy. Secondarily, we analyzed numerous clinical variables to determine potential predictors of positive findings on the imaging studies.

## Methods

### Study population

This was a retrospective, observational study of a cohort of 59 patients diagnosed with PCa who underwent 18F-FCH PET/CT at the Nuclear Medicine Department of the Hospital Universitario Quiron in Madrid between November 2011 and July 2015. Of these 59 patients, 21 were excluded from the study due to the following: staging 18F-CH PET/CT performed prior to the initial treatment (*n* = 5); biochemical failure after radical radiotherapy (*n* = 10); missing mpMRI (*n* = 3); lost to follow-up (*n* = 3). Therefore, all patients who presented a biochemical recurrence (defined as two consecutive elevations of PSA >0.2 ng/mL) after RP and also underwent mpMRI without endorectal coil and 18F-CH PET/CT within a maximum of 30 days of each other were included in the study (*n* = 38). The PSA values assessed prior to mpMRI and PET/CT imaging were statistically not significantly different. Patients who received salvage RT and/or androgen-deprivation therapy (ADT) after RP (*n* = 11) were also included. The study was approved by our institution’s ethics committee.

### Multiparametric magnetic resonance imaging protocol

The mpMRI protocol has been described in detail elsewhere [3, 13, 14]. Briefly, a 3T MRI was used (Signa HDxt 3.0 T G.E. Healthcare; Milwaukee, WI, USA) with a gradient strength of 33 mT/m and a gradient slew rate of 120 T/m/s. An 8-channel surface coil was used (Torso phased array). Morphological imaging included T1- and T2-weighted sequences and functional studies included DWI and DCE imaging. The apparent diffusion coefficient (ADC) map was calculated while using DWI and b-values of 0 and 1000 s/mm2 were used. DCE imaging was performed with gadolinium contrast. For DCE, a qualitative review was performed and uptake patterns of relapse-suspected lesions were classified as follows: type 1 curve (slow and progressive uptake), type 2 curve (initial uptake followed by a plateau), or type 3 curve (intense initial uptake followed by washout).

### 18F-choline PET/CT protocol

Patient preparation for PET-CT consisted of 4–6 h of fasting. All patients received an intravenous injection of 18F-fluoromethylcholine (4 MBq/kg) supplied by the Instituto Tecnologico PET (ITP) in Madrid. Whole body scan was started 60–90 min after injection of 18F-CH. Early dynamic images were not obtained. We just acquired late imaging. The whole-body acquisition was performed in the three dimensional mode, using 2 min per bed position from the base of the skull to the mid-thigh (six or seven bed positions). Images were reconstructed with a standard reconstruction ordered-subset expectation maximization iterative algorithm (two iterative steps) and reformatted into transverse, coronal and sagittal views. Diagnostic CT protocols (oral and intravenous contrasts) were applied. Furosemide was not used in this study. PET-CT scanning was performed using a Biograph 6 True Point HD LSO integrated device (Siemens Healthcare Molecular Imaging; Knoxville, Tennessee, USA) with an intrinsic axial resolution of 4.1 mm FWHM and iterative reconstruction. The CT scan (130–136 keV; 60–90 mAs) was taken in direct proportion to the patient’s weight, followed by a PET scan (performed in the same parameter range) for 3 min per bed (approximately 6–7 min). The PET data were processed with iterative reconstruction and converted into PET images (based on CT) with and without attenuation correction. Acquired images were examined on an LCD monitor as both attenuation-corrected and uncorrected multiplanar PET, CT, and PET/CT fusion cross-sections (maximum intensity projection = MIP), using the eSOFT software (Siemens, USA).

### Image analysis

A radiologist and a nuclear physician, both experts in uro-oncology, retrospectively reviewed the mpMRI and 18F-CH PET/CT images. These specialists performed the analysis independently of each other and both were blinded to outcomes. Radiological findings were scored from 1 to 3, as follows: a score of 1 indicated a negative finding (absence of recurrence), 2 was considered indeterminate, and 3 indicated positive findings (presence of recurrence). This same scoring approach was used to assess LR and bone and nodal metastases. All lesions with a score of 1 or 2 were considered negative for the purposes of the present study. The image analyses were performed as follows:

#### mpMRI

The diffusion sequence and the dynamic contrast-enhanced study were processed in a workstation (Advantage Workstation 4.3; GE Healthcare, Milwaukee, WI, USA). T2 and DWI sequences included the whole pelvis and iliac crests. DCE imaging was performed only in the prostate bed. LR was measured using the T2, DWI and DCE sequences while LNR and BM were assessed with T2 and DWI sequences. There is no validated scoring system to define a recurrence after RP on mpMRI [5]; consequently, we used the following mpMRI criteria for LR: presence of a soft tissue nodule on T2-weighted images in or around the prostatectomy bed in T2-weighted images; the presence of an area or hyperintense nodular lesion on the diffusion map, and a hypointense lesion on the ADC map with low ADC values and a DCE image showing an intense, early enhancement with plateau (type 2 curve) or posterior washing (type 3 curve). LR was positive considered when two or more sequences were abnormal. LNR were considered pathological when the short axis diameter was longer than 8 mm, the MRI signal was heterogeneous, and the contour was irregular. BM was considered pathological when abnormalities were evident on T2 and DWI.

#### 18F-CH PET/CT

18F-CH deposits with higher-than background activity not explained by physiological phenomena were considered positive. Semi-quantitative analysis of the abnormal radiotracer uptake was performed by using the maximum standardized uptake value (SUVmax). This value was obtained automatically.

### Salvage treatment

Patients who presented post-RP biochemical recurrence without visible evidence of a tumor on the imaging techniques were treated with rescue RT (70–74 Gy) to the prostate bed. In patients with LR, the dose was increased to 76 Gy. Patients with a pelvic LNR received RT to the pelvic lymph nodes (52.8 Gy at 1.6 Gy/fraction) and the prostatectomy bed (66 Gy at 2 Gy/fraction), together with a simultaneous integrated boost (SIB) to affected lymph nodes (72.6 Gy at 2.2 Gy/fraction). The pelvic BMs were also included within the RT treatment volume with SIB (72.6 Gy at 2.2 Gy/fraction). In patients who had been previously treated with prostate bed RT and later developed pelvic LNR and/or BM, stereotactic fractionated body radiotherapy (SBRT) was prescribed to this localization (excluding the pelvis) as follows: BM: 27–35 Gy in 3 or 5 fractions; LNR: 30–37.5 Gy in 3 or 5 fractions. In oligometastatic (<5 metastases) patients with distant metastases, each lesion was treated with SBRT. ADT was added to the RT prescription at the discretion of the treating physician. However, patients with multiple distant metastases were treated with ADT alone.

### Statistical analysis

Quantitative variables are given as medians with interquartile range (IQR) or as a mean ± standard deviation (SD). For qualitative variables, absolute and relative frequencies are given in percentages. The chi-square test was used to analyze qualitative variables. The student’s *T* test or the Mann–Whitney *U* Test were used, as appropriate, to analyze significant differences among the quantitative variables.

McNemar’s test was used to analyze differences between mpMRI and 18F-CH PET/CT in detection rates. A per patient analysis was performed. A Venn’s diagram was used to show the distribution of LR and LNR [15].

A univariate/multivariate logistic regression analysis was performed to identify independent variables associated with the imaging findings. The statistical analysis was performed using SPSS, v. 21.0 (IBM Corp; Armonk, NY; USA), with *p* < 0.05 considered significant for all analyses.

## Results

The clinical characteristics and the variables related to the treatment of the 38 patients included in this study are detailed in Table 1. Treatment before 18F-CH PET/CT and mpMRI imaging consisted of the following: in 27 patients (71.1%), RP alone; in 4 patients (10.5%), RP plus ADT; and in 7 patients (18.4%), RP plus salvage RT. The median time relapsed from RP to mpMRI and 18F-CH PET/CT was 27.5 months [54]. Median age was 62.9 years [7.2]. In 13 cases (34.2%), the tumour stage was pT3. The Gleason score was >7 in 10 (26.3%) patients. Sixteen patients (42.1%) had positive surgical margins. The median PSA value prior to imaging was 0.9 ng/mL (interquartile range 0.4–2.2).

**Table 1:** Clinical, pathologic and treatment related characteristics

	ALL
	*n* = 38
Age, years	62,9 ± 7,2
Preoperative PSA, ng/mL	7,4 [9,8]
Pathologic T stage	
T2	25 (65,8)
T3	13 (34,2)
Pathologic N stage	
N0	16 (42,1)
Nx	22 (57,9)
Pathologic Gleason score	
≤ 7	28 (73,7)
> 7	10 (26,3)
Positive surgical margin	
Yes	16 (42,1)
No	22 (57,9)
Perineural Invasion	
Yes	17 (44,7)
No	21 (55,3)
Lymphatic vessel invasion	
Yes	4 (10,5)
No	34 (89,5)
PSA levels, ng/mL	
Post radical prostatectomy	0,1 [0,3]
On day of biochemical failure	0,4 [0,7]
On day of choline PET/CT and mpMRI	0,9 [1,8]
Lowest PSA level after surgery	0,1 [0.3]
Treatment before mpMRI/choline PET/CT	
Radical prostatetcomy only	27 (71,1)
Radical prostatectomy and hormonotherapy	4 (10,5)
Radical prostatectomy and radiotherapy	7 (18,4)
Time from prostatectomy, months	
To first PSA recurrence	10,5 [22,3]
To mpMRI/PET/CT	27,5 [54,0]
PSA doubling time, months	4,5 [8,3]

Mean ± standard deviation; Median [interquartile range]; n (%)

**Fig. 1:**
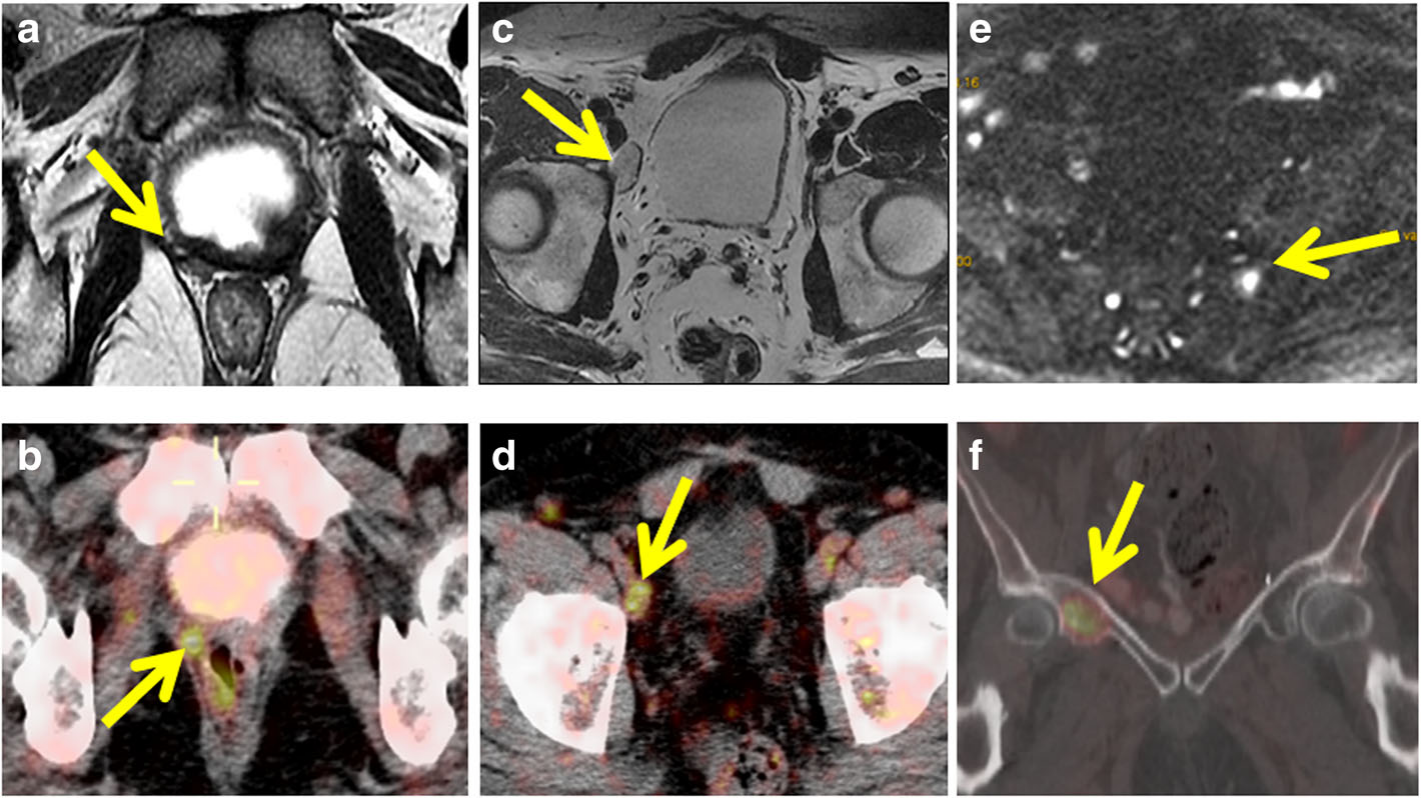
Representative images of different pelvic tumor recurrences detected by mpMRI and 18F-CH PET/CT (*arrows*). **a** Local recurrence in axial T2-weighted MRI and **b** local recurrence in 18F-CH PET/CT. **c** Right external iliac lymph node detected by axial T2-weighted MRI. **d** 18F-CH PET/CT image at corresponding level demonstrates choline-avid right external iliac lymph node. **e** Axial Diffusion Weighted Imaging (DWI) of bone metastases in left sacrum (*arrow*) and; **f** 18F-CH PET/CT shows a hypermetabolic bone metastases in right acetabulum

Separately, mpMRI and 18F-CH PET/CT were positive for recurrence in 55.2% (21/38 patients) and 52.6% (20/38 patients) of cases, respectively. When combined findings were considered, 65.7% (25/38 patients) of cases were positive for recurrence (Fig. 1). The combination of MRI and choline PET/CT resulted in a higher detection rate for LR versus choline PET/CT alone (34.2% vs 18.4%, *p* = 0.04). No significant differences were observed between 18F-CH PET/CT imaging and mpMRI imaging in terms of their detection rates for LR, LNR and BM, which were, respectively, as follows: 18.4% vs 31.6% (*p* = 0.12), 31.6% vs 26.3% (*p* = 0.50), and 7.9% vs 10.5% (*p* = 1.00) (Table 2). Distance metastases were detected by 18F-CH PET/CT in 5 patients (3 patients with mediastinal lymph node metastases, 1 with a supraclavicular nodal metastasis, and 1 with retroperitoneal metastases). Overall, the radiological findings of both tests disagreed in 10 cases.

**Table 2:**
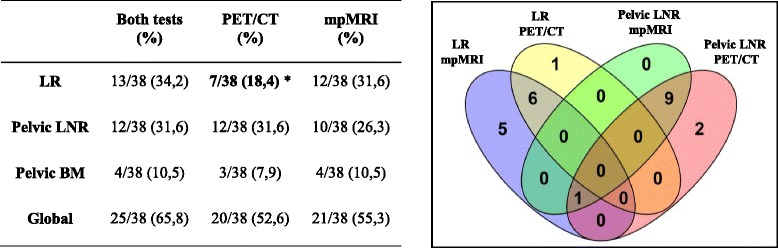
Comparison of the detection rate of mpMRI versus 18F-Choline PET/TC versus Both tests in the diagnosis of tumor recurrences in patients with biochemical relapse following radical prostactectomy. The right panel shows a Venn’s Diagram of the distribution of the most frequent recurrences of these patients: LR and LNR

*LR* local recurrence, *LNR* lymph node recurrence, *BM* bone metastases

Statistically significant value, *p < 0.05 vs. Both test are in bold

The initially planned salvage treatment was modified in 22 patients (57.8%) due to mpMRI and/or 18F-CH PET/CT findings (Table 3).

**Table 3:** Modification of salvage treatment after radiological findings of mpMRI and/or 18F-CH PET/CT

N	Initial treatment	Salvage treatment, initially planned BEFORE imaging	Radiological findings of mpMRI and/or 18F-CH PET/CT	Salvage treatment, planned AFTER imaging
8	Radical prostatectomy	RT on prostate bed	LR	Boost on tumor bed
1	Radical prostatectomy	RT on prostate bed	Pelvic LNR + one pelvic BM	RT on pelvis (including LNR and pelvic BM with SIB) + ADT
1	Radical prostatectomy and ADT	RT on prostate bed	LR+ One pelvic BM	RT on bed + BM + ADT
8	Radical prostatectomy	RT on prostate bed	Pelvic LNR	RT on pelvis (including LNR with SIB) + ADT
1	Radical prostatectomy and adjuvant radiotherapy	ADT	Pelvic and retroperitoneal LNR + one pelvic BM	SBRT on affected node and BM + ADT
3	Radical prostatectomy and ADT	RT on prostate bed	LR + Pelvic LNR	RT on pelvis (including LR and LNR with SIB) + ADT

### Clinical variables associated with the radiological findings

The 21 patients with a positive mpMRI had significantly higher PSA values prior to imaging than patients with a negative mpMRI (Table 4). Patients with a positive mpMRI had a significantly higher median PSA at biochemical failure compared to patients with negative mpMRI findings (0.9 [1.2] ng/mL versus 0.4 [0.1] ng/mL, *p* = 0.005). PSA values were significantly associated with a positive mpMRI (odds ratio [OR]: 30.9; 95% confidence interval [CI]: 1.5–635.8) (Table 5).

**Table 4:** Comparison of the clinical variables of patients with positive and negative mpMRI and 18F-CH PET/CT

	mpMRI			18F-CH PET/CT		
	Positive *n* = 21	Negative/Uncertain *n* = 17	*P* value	Positive *n* = 20	Negative/Uncertain *n* = 18	*P* value
Age, years	63,2 ± 7,2	62,5 ± 7,4	0,767	63,4 ± 7,6	62,4 ± 6,8	0,703
Preoperative PSA, ng/mL	7,7 [14,0]	7,0 [8,4]	0,453	7,4 [9,1]	7,5 [12,1]	0,65
Pathologic T stage (%)						
T2	11 (44,0)	14 (56,0)	0,086	11 (44,0)	14 (56,0)	0,182
T3	10 (76,9)	3 (23,1)		9 (69,2)	4 (30,8)	
Pathologic N stage, n (%)						
N0	9 (56,3)	7 (43,8)	0,917	10 (62,5)	6 (37,5)	0,299
Nx	12 (54,5)	10 (45,5)		10 (45,5)	12 (54,5)	
Pathologic Gleason score, n (%)						
≤ 7	13 (46,4)	15 (53,6)	0,136	11 (39,3)	17 (60,7)	**0,009**
> 7	8 (80,0)	2 (20,0)		9 (90,0)	1 (10,0)	
Positive surgical margin, n (%)						
Yes	8 (50,0)	8 (50,0)	0,578	6 (37,5)	10 (62,5)	0,111
No	13 (59,1)	9 (40,9)		14 (63,6)	8 (36,4)	
Perineural Invasion, n (%)						
Yes	10 (58,8)	7 (41,2)	0,691	9 (52,9)	8 (47,1)	0,973
No	11 (52,4)	10 (47,6)		11 (52,4)	10 (47,6)	
Lymphatic vessel invasion, n (%)						
Yes	2 (50,0)	2 (50,0)	1,000	2 (50,0)	2 (50,0)	1,000
No	19 (55,9)	15 (44,1)		18 (52,9)	16 (47,1)	
PSA levels, ng/mL						
Post radical prostatectomy	0,2 [1,4]	0,0 [0,1]	**0,006**	0,2 [1,3]	0,0 [0,1]	**0,007**
On day of biochemical failure	0,9 [1,2]	0,4 [0,1]	**0,005**	0,7 [1,1]	0,4 [0,1]	**0,009**
On day of CH PET/CT and mpMRI	2,0 [1,9]	0,4 [1,0]	**0,024**	2,0 [1,7]	0,4 [0,7]	**0,019**
Lowest PSA level after surgery	0,2 [1,4]	0,0 [0,1]	**0,012**	0,2 [1,2]	0,0 [0,1]	**0,003**
Treatment before mpMRI/18F-CH PET, n (%)						
Radical prostatectomy only	14 (51,9)	13 (48,1)	0,508	15 (55,6)	12 (44,4)	0,572
Prostatectomy and HT or RT	7 (63,6)	4 (36,4)		5 (45,5)	6 (54,5)	
Time from prostatectomy, months						
To first PSA recurrence	5,0 [36,0]	16,0 [18,5]	0,075	5,5 [21,0]	16,5 [25,0]	0,079
To mpMRI/PET/CT	31,0 [55,5]	27,0 [44,5]	0,596	10,5 [36,0]	32,0 [48,0]	0,187
PSA doubling time, months	4,0 [9,5]	5,8 [8,5]	0,634	3,1 [5,3]	8,5 [9,5]	0,135

Mean ± standard deviation; Median [interquartile range]

Statistically significant value, *p < 0.05 *are in bold

**Table 5:** Univariate analysis of the clinic characteristics associated to the positivity of mpMRI and 18F-CH PET/CT

	mpMRI		18F-CH PET/CT	
	OR (95% CI)	*p* value	OR (95% CI)	*p* value
Age, years	1.01 (0.93–1.10)	0.759	1.02 (0.93–1.12)	0.694
Preoperative PSA, ng/mL	1.05 (0.95–1.15)	0.344	0.98 (0.89–1.07)	0.630
Pathologic T stage, T3	4.24 (0.94–19.26)	0.061	2.86 (0.69–11.82)	0.146
Pathologic N stage, Nx	0.93 (0.26–3.41)	0.917	0.50 (0.13–1.86)	0.301
Pathologic Gleason score, > 7	4.62 (0.83–25.73)	0.081	13.91 (1.54–125.63)	**0.019**
Positive surgical margin	0.69 (0.19–2.53)	0.578	0.34 (0.09–1.30)	0.116
Perineural Invasion	1.30 (0.36–4.72)	0.691	1.02 (0.28–3.68)	0.973
PSA levels, ng/mL				
Post radical prostatectomy	20.66 (0.45–964.07	0.121	2.17 (0.71–6.63)	0.172
On day of biochemical failure	30.90 (1.50–635.84)	**0.026**	2.48 (0.76–8.15)	0.135
On day of MRI-PET/CT	1.62 (0.88–2.98)	0.122	1.64 (0.90–2.99)	0.110
Lowest PSA level after surgery	16.77 (0.530–531.40)	0.110	2.03 (0.72–5.69)	0.180
Treatment before mpMRI/choline PET/CT				
Radical prostatectomy and hormonotherapy or radiotherapy	1.63 (0.38–6.87)	0.509	0.67 (0.16–2.73)	0.573
Time from prostatectomy				
To first PSA recurrence	0.99 (0.95–1.03)	0.568	0.98 (0.94–1.01)	0.217
To MRI-PET/CT	1.00 (0.98–1.02)	0.807	0.99 (0.97–1.01)	0.203
PSA doubling time, months	1.02 (0.92–1.12)	0.772	0.94 (0.85–1.05)	0.271

Statistically significant value, *p* < 0.05 are in bold

The 20 patients with a positive 18F-CH PET/CT presented significantly higher PSA values prior to imaging than patients with a negative PET/CT (Table 4). Additionally, the Gleason score and subsequent PET/CT findings were significantly associated: of the 10 patients with a Gleason score >7, nine (90%) had a positive PET/CT; however, of the 28 patients with a Gleason score ≤7, only 11 (39.3%) had a positive PET/CT. Consequently, Gleason score > 7 was significantly associated with a positive PET/CT (OR: 13.9; 95% CI: 1.5–125.6) (Table 5).

LR occurred in 13 patients. Additional file 1 shows the distribution of clinical variables in this subgroup. On the multivariate analysis, a significant association was found between PSADT (OR: 1.3; 95% CI: 1.0–1.7), T stage (OR: 21.1;95% CI:1.6–272.1), and the presence of LR (Additional file 2).

A total of 12 patients presented LNR. In patients with a Gleason score > 7, six of 10 patients (60%) developed a LNR versus only six of the 28 patients (21.4%) with a Gleason score ≤7, a significant difference (*p* = 0.04). PSA values (first PSA after surgery, PSA at biochemical failure, and nadir PSA after RP) were all significantly higher in patients who developed LNR. PSADT, the time elapsed from RP to first biochemical recurrence, and time elapsed from surgery to MRI and PET-CT, were all significantly shorter in patients who developed LNR (see Additional file 3). The univariate analysis showed a significant association between Gleason score (OR: 5.5; 95% CI: 1.2–26.0), PSADT (OR: 0.74; 95% CI: 0.56–0.97) and the presence of LNR (see Additional file 4). On the multivariate analysis, no associations were observed between any of the clinical variables and LNR.

## Discussion

To our knowledge, this is the first study to compare 3T-mpMRI without endorectal coil to 18F-CH PET/CT to determine their relative capacity to detect pelvic tumor recurrences following RP. Although the detection rate of LR, LNR and BM was similar in both imaging techniques, the combination of mpMRI and 18F-CH PET/CT was superior to PET/CT alone in the detection of LR.

Few studies have compared mpMRI to choline PET/CT in the context of biochemical relapse after RP. Kitajima et al. retrospectively compared 11C-choline PET/CT to 1.5T and 3T mpMRI with endorectal coil to detect pelvic recurrences after RP in 115 PCa patients [8]. In that study, mpMR imaging with endorectal coil was superior to PET/CT in the detection of LR, but PET/CT was superior for detecting LNR; both were equally excellent for pelvic BM []. Panebianco et al. compared 3T MRI with endorectal coil to 18F-CH PET/CT in 84 patients with PCa recurrence [7], finding a diagnostic accuracy of MRI for LR higher than that of PET/CT.

In recent years, a new generation of targeted tracers for PET imaging, primarily PSMA, has shown promising results, with a better detection rate than MRI or choline imaging, even in patients with low PSA levels (≤0.5 ng/mL) [3]. However, we are still awaiting the results of validation studies currently in progress [16].

We found that, the tumor detection rate in our patient cohort was high: nearly two-thirds for combined MRI and PET/CT versus approximately half for both MRI and PET/CT alone. In addition, the combination of the imaging techniques versus choline PET/CT alone achieved a significantly higher detection rate for LR. It is worth highlighting the increasing clinical and diagnostic role of the integrated PET/MRI scanner, an advanced technique that combines the anatomical and functional information provided by MRI with the higher specificity of PET [17]. This hybrid PET-MRI system has shown promising results in terms of tumor detection compared to MRI or PET alone [17]. For this reason, the high detection rate for MRI and choline PET/CT allows us to define the site of recurrence, assess for metastatic disease, and personalize salvage therapy. Thus, the use of combined data in our study had an important impact on salvage RT treatment decisions, leading us to change the therapeutic approach in nearly 60% of cases.

Salvage RT is the primary treatment option in patients who suffer a relapse after RP. Although the most common approach is to irradiate the surgical bed (even when there is no radiological or histological evidence of disease), various questions remain unresolved with regard to optimal target volume definition and RT doses [16]. Of the 38 patients in our study, 13 (34.2%) developed LR. In 8 of these patients, we were able to escalate the RT dose to 76 Gy at the recurrence site. This is important because the dose needed to achieve biochemical control can vary depending on whether the recurrence is micro- or macroscopic [18]. Moreover, when the imaging studies show no evidence of recurrence, radiation oncologists define the prostate bed and clinical target volume (CTV) blindly, based on recommendations published in clinical guidelines [19]. However, in some cases, following these guidelines can result in the tumor being excluded from the CTV [20]. Similarly, the decision to irradiate the prostate bed or the whole pelvis will depend on the localization of the recurrence, which can be local or loco-regional.

Although metastatic PCa has traditionally been treated with ADT alone, the recent increase of metastases-directed therapy in oligometastatic patients, has made it even more important to accurate detect the presence of LNR and BM after RP in order to properly manage these patients [21]. In our study, oligometastatic lesions (bone and/or lymph nodes) were detected outside of the prostate bed in nearly 37% of patients. In these cases, the initial salvage RT plan to the prostate bed would have been completely ineffective. For this reason, the definitive treatment included metastases-directed therapy with RT and ADT. Although it is true that the optimal therapy in patients with nodal or pelvic bone involvement remains unclear, a recent review of studies that assessed surgical and RT treatment of oligometastatic PCa concluded that, in general, both of these treatment approaches can achieve good outcomes in terms of disease control and increased survival, with only limited toxicity [21].

In terms of the association between clinical variables and the findings of the imaging tests, several points are worth highlighting. Patients with positive MRI and PET/CT findings had PSA values that were significantly greater than those observed in patients with negative findings on the imaging tests. In fact, although we only found an association between PSA values at the time of biochemical failure and MRI positivity, many studies have reported an association between PSA and choline PET/CT [22]. Thus, after RP, the optimal PSA cut-off level for choline PET/CT analysis seems to be between 1 and 2 ng/mL [23], while for MRI, the corresponding value appears to range from 0.3 to 0.54 ng/mL [5]. In addition to the PSA value, several studies have reported that PSA kinetics (PSADT, PSA velocity) are strong predictors for positive PET and MRI findings, even in patients with low PSA values [4, 5, 23]. We found that PSADT was significantly associated with the type of pelvic recurrence: patients with a higher PSADT had an increased probability of local recurrence. Hernández et al. demonstrated a significantly shorter PSADT in patients with LNR [5]. In the other hand, the Gleason score should be considered when evaluating the utility of 18F-CH PET/CT as it has been related to the PET/CT positivity [24]. T staging was also significantly associated with LR: patients with extracapsular involvement (T3) had an increased probability of developing LR, a finding that is congruent with the beneficial effect of adjuvant RT in the patient subgroup with extracapsular invasion or seminal vesicle involvement [23]. In short, all of these variables should be taken into account when re-staging patients who develop biochemical recurrence after RP in order to select the most appropriate salvage therapy.

Our study has several limitations. First, this was a retrospective analysis with a small sample size. Second, there was some patient selection bias, as evidenced by the fact that most patients who underwent mpMRI and 18F-CH PET/CT had positive radiological findings. This can be attributed to various factors, as follows: pre-imaging PSA values were relatively high in the entire cohort, the time to biochemical failure was short, and the PSADT values low. All of these clinical characteristics are indicative of highly aggressive disease with a high probability of tumor recurrence that are detectable on both imaging tests. Third, the specific choline PET/CT protocol and the radiotracer used in this study could have influenced the quality of the images and thus their interpretation. However, a recent metanalysis concluded that (11) C-choline and 18F-CH and the different acquisition protocols had no significant impact on the detection rate [25]. Fourth, the MRI and PET images were acquired in separate imaging sessions and this may have affected the detection rate compared to the hybrid PET-MRI system, as mentioned before. Fifth, we did not use endorectal coil and this may have influenced the reliability of the MRI images [26]. Nevertheless, in patients with tumor recurrence after RP, the tumor detection rate using a 3T MRI seems not to be influenced by the type of coil [4]. Lastly, in our study 4 patients received ADT before MRI and PET/CT, which could have a limited uptake on the choline PET/CT and caused morphological changes and alterations in the parameters of the DWI and DCE on the MRI [27, 28]. However, data from our study and others [7] show that this does not directly influence the comparative analysis. In fact, in three of these four patients the recurrence was identified by both imaging techniques.

The strength of the study is the blinded evaluation of all images at the same institution by two different experts (an uro-radiologist and a nuclear medicine specialist). In addition, this is the first study to compare 3T mpMRI without endorectal coil to 18F-CH PET/CT in this context.

## Conclusion

Multiparametric MRI and 18F-CH PET/CT yield similar detection rates for LR, LNR and pelvic BM. The combination of both imaging techniques provides a better LR detection rate after RP versus choline PET/CT alone. The initially planned salvage treatment was modified in 57.8% of patients due to both imaging techniques findings. In addition to PSA values, Gleason score, T stage, and PSADT may provide valuable data to identify those patients that are most likely to benefit from undergoing both imaging procedures.

## Additional files

Additional file 1: Comparison of the clinical variables of patients with positive and negative local recurrence. (DOC 69 kb)
Additional file 2: Multivariate analysis of the clinical characteristics associated to Local Recurrence. (DOC 31 kb)
Additional file 3: Comparison of the clinical variables of patients with positive and negative lymph node recurrence. (DOC 71 kb)
Additional file 4: Univariate analysis of the clinic characteristics associated to the Local and Lymph Node recurrence. (DOC 55 kb)

## Abbreviations

18F-CH PET/CT: 18-fluorocholine positron emission tomography/computed tomography
ADC: Apparent diffusion coefficient
ADT: Androgen-deprivation therapy
BM: Bone metastases
DCE: Dynamic contrast enhanced
DWI: Diffusion weighted imaging
EAU: European Association of Urology
IQR: Interquartile range
ITP: Instituto Tecnológico PET
LNR: Lymph node recurrence
LR: Local recurrences
mpMRI: Multiparametric magnetic resonance imaging
PSADT: PSA doubling time
RP: Radical prostatectomy
RT: Radiotherapy
SBRT: Stereotactic fractioned body radiotherapy
SD: Standard deviation
SIB: Simultaneous integrated boost
SUVmax: Maximum standardized uptake value
