# Supplementary material for: Evaluation of tumor recurrences after radical prostatectomy using 18F-Choline PET/CT and 3T multiparametric MRI without endorectal coil: a single center experience
Source: Cancer Imaging. 2016 Dec 7;16:42. doi: 10.1186/s40644-016-0099-8 (PMC5142428; doi:10.1186/s40644-016-0099-8)
Supplement: Additional file 1: — Comparison of the clinical variables of patients with positive and negative local recurrence. (DOC 69 kb) [file 40644_2016_99_MOESM1_ESM.doc]

|  | **TOTAL  n=38** | **Positive  n=13** | **Negative / Uncertain  n=25** | **p-value** |
| --- | --- | --- | --- | --- |
| **Age, years** | 62,9 ± 7,2 | 65,3 ± 6,7 | 61,7 ± 7,2 | 0,142 |
| **Preoperative PSA, ng/mL** | 7,4 [9,8] | 6,4 [9,8] | 8,2 [10,5] | 0,488 |
| **Pathologic T stage (%)** |  |  |  |  |
| T2 | 25 (65,8) | 6 (24,0) | 19 (76,0) | 0,084 |
| T3 | 13 (34,2) | 7 (53,8) | 6 (46,2) |
| **Pathologic N stage, n (%)** |  |  |  |  |
| N0 | 16 (42,1) | 6 (37,5) | 10 (62,5) | 0,715 |
| Nx | 22 (57,9) | 7 (31,8) | 15 (68,2) |
| **Pathologic Gleason score, n (%)** |  |  |  |  |
| ≤ 7 | 28 (73,7) | 9 (32,1) | 19 (67,9) | 0,709 |
| > 7 | 10 (26,3) | 4 (40,0) | 6 (60,0) |
| **Positive surgical margin, n (%)** |  |  |  |  |
| Yes | 16 (42,1) | 7 (43,8) | 9 (56,3) | 0,290 |
| No | 22 (57,9) | 6 (27,3) | 16 (72,7) |
| **Perineural Invasion, n (%)** |  |  |  |  |
| Yes | 17 (44,7) | 6 (35,3) | 11 (64,7) | 0,899 |
| No | 21 (55,3) | 7 (33,3) | 14 (66,7) |
| **Lymphatic vessel invasion, n (%)** |  |  |  |  |
| Yes | 4 (10,5) | 3 (75,0) | 1 (25,0) | 0,107 |
| No | 34 (89,5) | 10 (29,4) | 24 (70,6) |
| **PSA levels, ng/mL** |  |  |  |  |
| Post radical prostatectomy | 0,1 [0,3] | 0,1 [1,0] | 0,0 [0,2] | 0,170 |
| On day of biochemical failure | 0,4 [0,7] | 0,5 [1,1] | 0,4 [0,4] | 0,248 |
| On day of choline PET/CT and mpMRI | 0,9 [1,8] | 2,1 [2,2] | 0,6 [1,6] | 0,074 |
| Lowest PSA level after surgery | 0,1 [0,3] | 0,1 [1,0] | 0,0 [0,2] | 0,238 |
| **Treatment before mpMRI/18F-Choline PET, n (%)** |  |  |  |  |
| Radical prostatectomy only | 27 (71,1) | 8 (29,6) | 19 (70,4) | 0,351 |
| Prostatectomy and hormonotherapy or radiotherapy | 11 (28,9) | 5 (45,5) | 6 (54,5) |
| **Time from prostatectomy, months** |  |  |  |  |
| To first PSA recurrence | 17,8 ± 17,4 | 18,2 ± 20,2 | 17,6 ± 16,2 | 0,600 |
| To mpMRI/PET/CT | 27,5 [54,0] | 31,0 [65,5] | 27,0 [39,5] | 0,711 |
| **PSA doubling time, months** | 4,5 [8,3] | 6,0 [12,0] | 4,0 [8,0] | 0,175 |

**Additional file 3** Comparison of the clinical variables of patients with positive and negative local recurrence.
