# Supplementary material for: Evaluation of tumor recurrences after radical prostatectomy using 18F-Choline PET/CT and 3T multiparametric MRI without endorectal coil: a single center experience
Source: Cancer Imaging. 2016 Dec 7;16:42. doi: 10.1186/s40644-016-0099-8 (PMC5142428; doi:10.1186/s40644-016-0099-8)
Supplement: Additional file 2: — Multivariate analysis of the clinical characteristics associated to Local Recurrence. (DOC 31 kb) [file 40644_2016_99_MOESM2_ESM.doc]

**Additional file 2** Multivariate analysis of the clinical characteristics associated to Local Recurrence.

| Variable | OR (95% CI) | p value |
| --- | --- | --- |
| | **Pathologic T stage ,** T3 | pT | | --- | --- | | **PSA doubling time, months** |  | | | 21.07 (1.63-272.11) | | --- | | 1.36 (1.02-1.68) | | | **0.020** | | --- | | **0.037** | |
