# Supplementary material for: Evaluation of tumor recurrences after radical prostatectomy using 18F-Choline PET/CT and 3T multiparametric MRI without endorectal coil: a single center experience
Source: Cancer Imaging. 2016 Dec 7;16:42. doi: 10.1186/s40644-016-0099-8 (PMC5142428; doi:10.1186/s40644-016-0099-8)
Supplement: Additional file 3: — Comparison of the clinical variables of patients with positive and negative lymph node recurrence. (DOC 71 kb) [file 40644_2016_99_MOESM3_ESM.doc]

|  | **TOTAL  n=38** | **Positive  n=12** | **Negative / Uncertain  n=26** | **p-value** |
| --- | --- | --- | --- | --- |
| **Age, years** | 62,9 ± 7,2 | 60,8 ± 7,0 | 63,9 ± 7,2 | 0,210 |
| **Preoperative PSA, ng/mL** | 7,4 [9,8] | 12,3 [14,7] | 6,6 [7,0] | **0,066** |
| **Pathologic T stage (%)** |  |  |  |  |
| T2 | 25 (65,8) | 6 (24,0) | 19 (76,0) | 0,270 |
| T3 | 13 (34,2) | 6 (46,2) | 7 (53,8) |
| **Pathologic N stage, n (%)** |  |  |  |  |
| N0 | 16 (42,1) | 6 (37,5) | 10 (62,5) | 0,503 |
| Nx | 22 (57,9) | 6 (27,3) | 16 (72,7) |
| **Pathologic Gleason score, n (%)** |  |  |  |  |
| ≤ 7 | 28 (73,7) | 6 (21,4) | 22 (78,6) | **0,045** |
| > 7 | 10 (26,3) | 6 (60,0) | 4 (40,0) |
| **Positive surgical margin, n (%)** |  |  |  |  |
| Yes | 16 (42,1) | 3 (18,8) | 13 (81,3) | 0,147 |
| No | 22 (57,9) | 9 (40,9) | 13 (59,1) |
| **Perineural Invasion, n (%)** |  |  |  |  |
| Yes | 17 (44,7) | 7 (41,2) | 10 (58,8) | 0,252 |
| No | 21 (55,3) | 5 (23,8) | 16 (76,2) |
| **Lymphatic vessel invasion, n (%)** |  |  |  |  |
| Yes | 4 (10,5) | 0 (0,0) | 4 (100,0) | 0,287 |
| No | 34 (89,5) | 12 (35,3) | 22 (64,7) |
| **PSA levels, ng/mL** |  |  |  |  |
| Post radical prostatectomy | 0,1 [0,3] | 0,4 [1,3] | 0,0 [0,1] | **0,008** |
| On day of biochemical failure | 0,4 [0,7] | 0,9 [1,4] | 0,4 [0,3] | **0,016** |
| On day of choline PET/CT and mpMRI | 0,9 [1,8] | 1,3 [1,6] | 0,7 [2,0] | 0,593 |
| Lowest PSA level after surgery | 0,1 [0,3] | 0,4 [1,3] | 0,0 [0,1] | **0,011** |
| **Treatment before mpMRI/18F-Choline PET, n (%)** |  |  |  |  |
| Radical prostatectomy only | 27 (71,1) | 9 (33,3) | 18 (66,7) | 0,715 |
| Prostatectomy and hormonotherapy or radiotherapy | 11 (28,9) | 3 (27,3) | 8 (72,7) |
| **Time from prostatectomy, months** |  |  |  |  |
| To first PSA recurrence | 10,5 [22,3] | 4,0 [8,3] | 16,5 [25,0] | **0,031** |
| To mpMRI/PET/CT | 27,5 [54,0] | 7,5 [33,0] | 33,5 [53,0] | **0,025** |
| **PSA doubling time, months** | 4,5 [8,3] | 3,0 [0,8] | 8,0 [11,0] | **0,010** |

**Additional file 3.** Comparison of the clinical variables of patients with positive and negative lymph node recurrence. Statistically significant value, p < 0.05 are in bold.
