# Supplementary material for: Evaluation of tumor recurrences after radical prostatectomy using 18F-Choline PET/CT and 3T multiparametric MRI without endorectal coil: a single center experience
Source: Cancer Imaging. 2016 Dec 7;16:42. doi: 10.1186/s40644-016-0099-8 (PMC5142428; doi:10.1186/s40644-016-0099-8)
Supplement: Additional file 4: — Univariate analysis of the clinic characteristics associated to the Local and Lymph Node recurrence. (DOC 55 kb) [file 40644_2016_99_MOESM4_ESM.doc]

|  | **Local recurrence** | | **Lymph Node recurrence** | |  |
| --- | --- | --- | --- | --- | --- |
|  | OR (95% CI) | p value | OR (95% CI) | p value | |
| **Age, years** | 1.09 (0.98 – 1.19) | 0.144 | 0.94 (0.84 – 1.04) | 0.207 | |
| **Preoperative PSA, ng/mL** | 0.99 (0.90 – 1.15) | 0.794 | 1.08 (0.98 – 1.19) | 0.138 | |
| **Pathologic T stage ,** T3 | 3.69 (0.89 – 15.37) | 0.072 | 2.70 (0.65 – 11.29) | 0.170 | |
| **Pathologic N stage ,** Nx | 0.78 (0.20 – 3.01) | 0.716 | 0.63 (0.16 – 2.49) | 0.504 | |
| **Pathologic Gleason score ,**  > 7 | 1.41 (0.32 – 6.27) | 0.654 | 5.50 (1.16 – 26.02 | **0.032** | |
| **Positive surgical margin** | 2.07 (0.53 – 8.10) | 0.294 | 0.33 (0.07 – 1.52) | 0.156 | |
| **Perineural Invasion** | 1.09 (0.28 – 4.19) | 0.899 | 2.24 (0.56 – 9.02) | 0.257 | |
| **PSA levels, ng/mL** |  |  |  |  | |
| Post radical prostatectomy | 1.12 (0.63 – 1.98) | 0.700 | 2.34 (0.89 – 6.16) | 0.085 | |
| On day of biochemical failure | 1.25 (0.64 – 2.43) | 0.513 | 2.45 (0.90 – 6.65) | 0.078 | |
| On day of choline PET/CT and mpMRI | 1.39 (0.81 – 2.38) | 0.232 | 0.94 (0.71 – 1.23) | 0.627 | |
| Lowest PSA level after surgery | 1.26 (0.64 – 2.48) | 0.500 | 2.21 (0.91 – 5.39) | 0.082 | |
| **Treatment before mpMRI/choline PET/CT** |  |  |  |  | |
| Radical prostatectomy and hormonotherapy or radiotherapy | 1.98 (0.47 – 8.40) | 0.355 | 0.75 (0.16 – 3.5) | 0.716 | |
| **Time from prostatectomy** |  |  |  |  | |
| To first PSA recurrence | 1.00 (0.96 – 1.04) | 0.930 | 0.92 (0.91 – 1.01) | 0.115 | |
| To mpMRI-PET/CT | 1.01 (0.99 – 1.03) | 0.277 | 0.97 (0.94 – 1.00) | 0.054 | |
| **PSA doubling time, months** | 1.11 (0.99 – 1.24) | 0.086 | 0.74 (0.56 – 0.97) | **0.029** | |

**Additional file 4.** Univariate analysis of the clinic characteristics associated to the Local and Lymph Node recurrence. Statistically significant value, p<0.05 are in bold
